# From Event-Related Potential to Oscillations: Genetic Diathesis in Brain (Dys)Function and Alcohol Dependence

**Published:** 2008

**Authors:** Madhavi Rangaswamy, Bernice Porjesz

**Keywords:** Alcoholism, alcohol disorders, children of alcoholics, alcohol-related genetic factors, risk factors, genetic markers, brain function, brain wave, brain activity diagnosis, neuroimaging, electroencephalography (EEG), event-related potential (ERP), P3 amplitude, event-related oscillation (ERO)

Recording the brain’s electrical activity using electrodes placed on the individual’s scalp provides noninvasive sensitive measures of brain function in humans. Regardless of whether an individual receives sensory information or performs higher cognitive processes, the brain regions involved exhibit measurable electrical activity, and by recording this activity with numerous electrodes placed on different areas of the scalp, researchers can determine when and where in the brain information processing occurs. Two general approaches can be used to record these neuroelectric phenomena:

- The continuous electroencephalogram (EEG) records brain activity when the subject is at rest and not involved in a task. It reflects the sum of the random activity of thousands of neurons that have similar spatial orientation in the brain. This activity typically fluctuates in wave-like patterns, and depending on the frequency of these patterns, one distinguishes different brain waves called δ (frequency of 1 to 3 Hz), θ (frequency of 4 to 7 Hz), α (frequency of 8 to 12 Hz), β (frequency of 12 to 28 Hz), and γ (frequency of 28+ Hz) rhythms. Variations in the patterns of these brain waves can indicate the level of consciousness, psychological state, or presence of neurological disorders.
- Event-related potentials (ERPs) are recorded while the subject is performing a sensory or cognitive task. They reflect the summated activity of network ensembles active during the task and are characterized by a specific pattern called the waveform, which is composed of negative and positive deflections (i.e., waves). For example, a target stimulus detected amidst a series of other nontarget stimuli produces a positive wave around 300 milliseconds after the stimulus. This is known as the P300 or P3 response.

The main advantage of these techniques is that they provide millisecond-by-millisecond indices of brain function and therefore provide excellent temporal resolution. However, because these measurements typically record the activity of thousands of neurons spread out over a certain area, they provide less spatial resolution than many neuroimaging methods (e.g., magnetic resonance imaging). In recent years a third type of electrophysiological response, event-related oscillations (EROs) have been identified that may serve as a measure of cognitive functions and which, as described in this article, may serve as markers for alcoholism risk.

## Event-Related Oscillations: A Measure of Cognitive Functions

Until recently, ERPs were the basic electrophysiological indices of cognition that provided valuable insights into human brain processes. Substantial literature now indicates that some ERP features may arise from changes in the dynamics of ongoing EEG rhythms/oscillations of different frequency bands that reflect ongoing sensory and/or cognitive processes (Basar 1999; Basar et al. 1999; Buzsaki 2006). In other words, the EEG oscillations that are measured in a resting state become organized, amplified, and/or coupled during mental activity, or the network activity induced by an event or stimulus may trigger specific oscillatory responses, thus giving rise to an “evoked” (strongly locked to the stimulus/event) or “induced” (weakly associated with to the stimulus/event) rhythmicity (Basar 1980; Basar 1999; Makeig et al. 2002). These EROs can influence the timing of neural activity and coordinate synchronous activity in groups of active neurons (Fries 2005). Thus, EROs represent a basic mechanism of neural communication, providing links to associative and integrative brain functions. High-frequency (i.e., β, γ) EROs are implicated in short range communication, whereas low frequencies (i.e., δ, θ, and α) EROs are involved in longer-range communication between brain areas (von Stein and Sarnthein 2000).

## From P3 to Oscillations: New Markers for Alcoholism Risk

**Figure 2:**
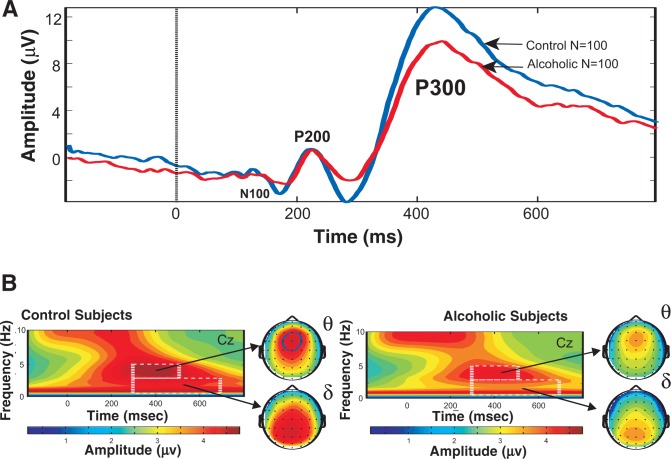
**A)** Grand mean event-related potential (ERP) at the central electrode (Cz) to visual oddball target processing, averaged from responses of 120 control subjects (blue trace) and 120 alcohol-dependent subjects (red trace). The curves show a reduced P3 (or P300) amplitude in alcoholics. Other prominent components of the waveform (P200, N100, and N200) are also labeled. **B)** Time x frequency plots for the target condition measured at the Cz electrode (central midline) location on the top of the head in 120 alcoholic (right panel) and 120 control (left panel) subjects. The headplots of the time-frequency regions of interest that underlie the duration of the P3 wave indicate that the peak power in θ band waves (4 to 5 Hz) during target processing (300–500 ms after the stimulus) is present in the anterior location on the scalp, whereas the δ band waves (1 to 3 Hz) peak power is seen in the posterior regions. Alcoholics have weaker responses than control subjects in both the θ and δ bands during the P3 response to target stimuli.

Abnormalities in EEG and ERP measures have been demonstrated to be good markers for certain neurological and psychiatric impairments as well as good indicators of risk status for certain conditions, including alcoholism. Research has consistently found that when compared with normal control subjects, the P3 component of the ERP is smaller (i.e., has a reduced amplitude) in alcoholics and children of alcoholics who are at increased risk of developing alcoholism but have not yet been exposed to alcohol (see figure 2) (Begleiter et al. 1984; for a review, see Porjesz et al. 2005). The lower P3 amplitudes, coupled with weaker and less well-organized sources (i.e., networks) in the brain, reflect inefficient allocation of resources during neural processing in alcoholics and offspring at risk. Low P3 amplitude also has been described in people with other disinhibitory disorders, such as substance abuse, antisocial personality disorder, conduct disorder, and attention deficit hyperactivity disorder (for a review, see Porjesz et al. 2005).

The P3 component is not a unitary phenomenon. This wave arises from activity in multiple sources that form a network within the brain. Important contributions from the frontal cortex (including anterior cingulate), parietal cortex, and hippocampus have been described (Ardekani et al. 2002; Kiehl and Liddle 2001; Menon et al. 1997). The energy in the P3 wave largely consists of contributions from θ and δ oscillations elicited during cognitive processing of stimuli. The energy of the δ oscillations is concentrated toward the posterior regions of the scalp, whereas the θ oscillations are more frontocentral or anterior (see figure 1) (Basar-Eroglu et al. 1992; Karakas et al. 2000*a*,*b*; Yordanova and Kolev 1996).

Recent studies found that the energy (power) in both δ and θ oscillations is reduced in alcoholics when compared with normal control subjects during the processing of target stimuli in a visual oddball paradigm^1^ (figure 2) (Jones et al. 2006*b*). These oscillations also were significantly lower in energy in adolescent offspring of alcoholics who are at high risk of developing dependence. Indeed, these reductions in brain oscillations were more sensitive than measurements of the P3 amplitude in discriminating between high- and low-risk offspring (Rangaswamy et al. 2007).

### Brain Oscillations as Endophenotypes

Phenotypes are observable characteristics or behaviors of an organism that are genetically determined, such as hair color (at least in animal models) or drinking behavior. Related phenomena are endophenotypes—traits or characteristics that are not a direct symptom of the condition under investigation (e.g., alcoholism) but which have been shown to be associated with the condition; for example, neurobiological characteristics such as reduced P3 amplitude have been noted in people with alcoholism and may be used as endophenotypes to identify people at risk for alcoholism. Brain oscillations also provide a rich source of useful endophenotypes for psychiatric genetics. They represent traits that are less complex than, for example, drinking behavior and likely are to be more directly related to the function of individual genes than the diagnosis of alcoholism. Moreover, as described above, brain oscillations can be used to differentiate affected and unaffected members of an affected family, including offspring at risk, providing a more direct connection with underlying biological vulnerability. Most importantly, these brain oscillations are highly heritable; thus, 76 percent of the variation in δ waves, 89 percent of the variation in θ and α waves, and 86 percent of the variation in β waves are genetically determined (van Beijsterveldt et al. 1996). This makes these brain oscillations highly useful for large genetic studies. For example, the Collaborative Study on the Genetics of Alcoholism (COGA) from its inception has utilized heritable and reliable neurophysiological traits that differentiate between alcoholics from densely affected alcoholic families and nonalcoholics from control families, as well as between high-risk unaffected offspring from the alcoholic families and low-risk offspring from control families, as endophenotypes to search for genes that are associated with the risk for alcohol dependence and related psychiatric disorders (Porjesz and Rangaswamy 2007).

**Figure 3:**
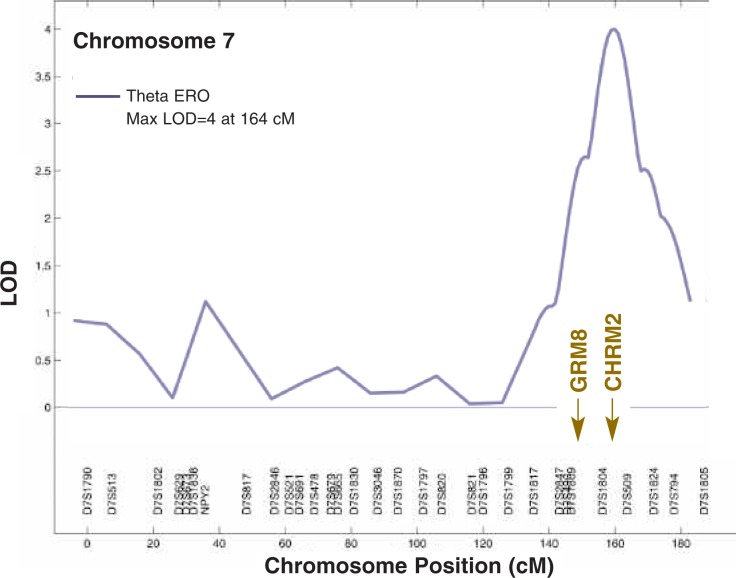
Results of linkage analyses demonstrating that specific gene variants on chromosome 7 are highly genetically linked with the development of θ wave (4–5 Hz) event-related oscillation (ERO) responses to a target stimulus. The region with the greatest linkage contains a gene encoding a receptor for the neurotransmitter acetylcholine (i.e., the cholinergic muscarinic receptor gene CHRM2) and a gene encoding a part of a glutamate receptor (i.e., the GRM8 gene). The dataset was derived from 1,337 individuals from 253 families. NOTE: The LOD (logarithm [base 10] of odds) is a measure of the degree of linkage between a given DNA region or gene and a specific trait.

EROs have served as successful endophenotypes in the search for genes involved in alcohol dependence and related disorders in COGA. For example, in a large sample from densely alcoholism-affected families, the frontal θ ERO underlying P3 in experiments using a visual odd-ball paradigm exhibited significant genetic linkage to a DNA region on chromosome 7 (Jones et al. 2006b). Two excellent candidate genes—CHRM2 and GRM8—are located in this region, both of which encode components of neurotransmitter receptors. CHRM2 encodes an acetylcholine receptor—the M2 muscarinic receptor—whereas GRM8 encodes the metabotropic glutamate receptor 8 that belongs to a family of G-protein–coupled receptors (see figure 3). Significant associations were observed between the frontal θ EROs that were generated when COGA participants responded to a target stimulus and certain variants (i.e., single-nucleotide polymorphisms [SNPs]) in the CHRM2 gene. Similar associations were reported for δ EROs recorded from parietal-occipital brain regions (Jones et al. 2004, 2006*a*). These findings implicate CHRM2 in the generation and modulation of these oscillations underlying the P3 response to target stimuli. They are supported by the observations that the generation of θ and δ oscillations depend on the level of activation of the M2 autoreceptors by acetylcholine (Fellous and Sejnowski 2000; Tiesinga et al. 2001). These receptors inhibit further acetylcholine release by the presynaptic cells, thus leading to inhibition of irrelevant networks. Other studies have suggested that acetylcholine plays a role in stimulus significance (Perry et al. 1999), selective attention (Mitrofanis and Guillery 1993), P3 generation, and modified memory performance (Callaway 1983; Dierks et al. 1994; Frodl-Bauch et al. 1999; Hammond et al. 1987; Potter et al. 2000). Thus, the genetic underpinnings of these oscillations likely stem from regulatory genes that control the neurochemical processes of the brain, thereby influencing neural function.

The major neurochemical components contributing to θ and δ rhythms and P3 involve strong GABAergic, cholinergic, and glutamatergic system interactions (Frodl-Bauch et al. 1999). Accordingly, researchers also investigated the association of the second gene identified as a candidate in the COGA linkage analyses, the GRM8 gene, with θ EROs in response to target stimuli. GRM8 modulates glutamate-mediated signal transmission in the brain by inhibiting glutamate release at the synapse. The analysis revealed that θ EROs in the frontal, central, and parietal regions of the cortex were significantly associated with several SNPs in the GRM8 gene (Chen et al. 2008). Moreover, several of the same SNPs also were significantly associated with a diagnosis of alcohol dependence using ICD–10 diagnostic criteria.

Recent evidence based on the COGA study indicates that the CHRM2 gene also is associated with a diagnosis of alcohol dependence and depression (Wang et al. 2004), comorbid alcohol and drug dependence (which reflects a more severe addiction profile) (Dick et al. 2007), and a spectrum of externalizing disorders (Dick et al. 2008). These findings have been replicated by other groups (Comings et al. 2002; Luo et al. 2005). Together, these results indicate that genes important for the expression of the endophenotype of brain oscillations can help researchers identify genes that increase the susceptibility for alcohol dependence and related disorders.

## Conclusions

Alcohol dependence and related disorders result from a complex interaction of genetic and environmental liabilities that change across development, with a greater impact of genetic factors in early-onset disorders. The use of quantitative brain oscillations provides a means to better understand the network dynamics of brain functions, and, by using these oscillations as endophenotypes, researchers can localize and characterize disease susceptibility genes more easily than if they have to rely on diagnostic categories (Begleiter and Porjesz 2006). The utility of electrophysiological measures as endophenotypes for studying the genetic risk of disinhibitory disorders, including alcoholism, is very promising.

## Footnotes

1 In a typical oddball experimental design, subjects must perform a task (e.g., press a button) when they see a rare target stimulus that appears 20 percent of the time (e.g., a red box) interspersed among a series of common nontarget stimuli that appear 80 percent of the time (e.g., a green box).

## References

[b1-arh-31-3-238] Ardekani BA, Choi SJ, Hossein-Zadeh GA (2002). Functional magnetic resonance imaging of brain activity in the visual oddball task. Brain Research Cognitive Brain Research.

[b2-arh-31-3-238] Basar E (1980). EEG-Brain Dynamics: Relation Between EEG and Brain Evoked Potentials.

[b3-arh-31-3-238] Basar E (1999). Brain Function and Oscillations. Vol. II: Integrative Brain Function, Neurophysiology and Cognitive Processes.

[b4-arh-31-3-238] Basar E, Basar-Eroglu C, Karakas S, Schurmann M (1999). Are cognitive processes manifested in event-related gamma, alpha, theta and delta oscillations in the EEG?. Neuroscience Letters.

[b5-arh-31-3-238] Basar-Eroglu C, Basar E, Demiralp T, Schurmann M (1992). P300-response: Possible psychophysiological correlates in delta and theta frequency channels: A review. International Journal of Psychophysiology.

[b6-arh-31-3-238] Begleiter H, Porjesz B (2006). Genetics of human brain oscillations. International Journal of Psychophysiology.

[b7-arh-31-3-238] Begleiter H, Porjesz B, Bihari B, Kissin B (1984). Event-related brain potentials in boys at risk for alcoholism. Science.

[b8-arh-31-3-238] Buzsaki G (2006). Rhythms of the Brain.

[b9-arh-31-3-238] Callaway E (1983). Presidential address, 1982: The pharmacology of human information processing. Psychophysiology.

[b10-arh-31-3-238] Chen ACH, Tang Y, Rangaswamy M (2008). Association of single nucleotide polymorphisms in a glutamate receptor gene (GRM8) with theta power of event-related oscillations and alcohol dependence. American Journal of Medical Genetics. Part B, Neuropsychiatric Genetics.

[b11-arh-31-3-238] Comings DE, Wu S, Rostamkhani M (2002). Association of the muscarinic cholinergic 2 receptor (CHRM2) gene with major depression in women. American Journal of Medical Genetics.

[b12-arh-31-3-238] Dick DM, Agrawal A, Wang JC (2007). Alcohol dependence with comorbid drug dependence: Genetic and phenotypic associations suggest a more severe form of the disorder with stronger genetic contribution to risk. Addiction.

[b13-arh-31-3-238] Dick DM, Aliev F, Wang JC (2008). Using dimensional models of externalizing psychopathology to aid in gene identification. Archives of General Psychiatry.

[b14-arh-31-3-238] Dierks T, Frolich L, Ihl R, Maurer K (1994). Event-related potentials and psychopharmacology: Cholinergic modulation of P300. Pharmacopsychiatry.

[b15-arh-31-3-238] Fellous JM, Sejnowski TJ (2000). Cholinergic induction of oscillations in the hippocampal slice in the slow (0.5–2 Hz), theta (5–12 Hz), and gamma (35–70 Hz) bands. Hippocampus.

[b16-arh-31-3-238] Frodl-Bauch T, Bottlender R, Hegerl U (1999). Neurochemical substrates and neuroanatomical generators of the event-related P300. Neuropsychobiology.

[b17-arh-31-3-238] Hammond EJ, Meador KJ, Aung-Din R, Wilder BJ (1987). Cholinergic modulation of human P3 event-related potentials. Neurology.

[b18-arh-31-3-238] Jones KA, Porjesz B, Almasy L (2004). Linkage and linkage disequilibrium of evoked EEG oscillations with CHRM2 receptor gene polymorphisms: Implications for human brain dynamics and cognition. International Journal of Psychophysiology.

[b19-arh-31-3-238] Jones KA, Porjesz B, Almasy L (2006a). A cholinergic receptor gene (CHRM2) affects event-related oscillations. Behavioral Genetics.

[b20-arh-31-3-238] Jones KA, Porjesz B, Chorlian D (2006b). S-transform time-frequency analysis of P300 reveals deficits in individuals diagnosed with alcoholism. Clinical Neurophysiology.

[b21-arh-31-3-238] Karakas S, Erzengin OU, Basar E (2000a). The genesis of human event-related responses explained through the theory of oscillatory neural assemblies. Neuroscience Letters.

[b22-arh-31-3-238] Karakas S, Erzengin OU, Basar E (2000b). A new strategy involving multiple cognitive paradigms demonstrates that ERP components are determined by the superposition of oscillatory responses. Clinical Neurophysiology.

[b23-arh-31-3-238] Kiehl KA, Liddle PF (2001). An event-related functional magnetic resonance imaging study of an auditory oddball task in schizophrenia. Schizophrenia Research.

[b24-arh-31-3-238] Luo X, Kranzler HR, Zuo L (2005). CHRM2 gene predisposes to alcohol dependence, drug dependence and affective disorders: Results from an extended case-control structured association study. Human Molecular Genetics.

[b25-arh-31-3-238] Makeig S, Westerfield M, Jung TP (2002). Dynamic brain sources of visual evoked responses. Science.

[b26-arh-31-3-238] Menon V, Ford JM, Lim KO (1997). Combined event-related fMRI and EEG evidence for temporal-parietal cortex activation during target detection. Neuroreport.

[b27-arh-31-3-238] Mitrofanis J, Guillery RW (1993). New views of the thalamic reticular nucleus in the adult and the developing brain. Trends in Neuroscience.

[b28-arh-31-3-238] Perry E, Walker M, Grace J, Perry R (1999). Acetylcholine in mind: A neurotransmitter correlate of consciousness?. Trends in Neuroscience.

[b29-arh-31-3-238] Porjesz B, Rangaswamy M (2007). Neurophysiological endophenotypes, CNS disinhibition, and risk for alcohol dependence and related disorders. Scientific World Journal.

[b30-arh-31-3-238] Porjesz B, Rangaswamy M, Kamarajan C (2005). The utility of neurophysiological markers in the study of alcoholism. Clinical Neurophysiology.

[b31-arh-31-3-238] Potter DD, Pickles CD, Roberts RC, Rugg MD (2000). Scopolamine impairs memory performance and reduces frontal but not parietal visual P3 amplitude. Biological Psychology.

[b32-arh-31-3-238] Rangaswamy M, Jones KA, Porjesz B (2007). Delta and theta oscillations as risk markers in adolescent offspring of alcoholics. International Journal of Psychophysiology.

[b33-arh-31-3-238] Tiesinga PH, Fellous JM, Jose JV, Sejnowski TJ (2001). Computational model of carbachol-induced delta, theta, and gamma oscillations in the hippocampus. Hippocampus.

[b34-arh-31-3-238] Van Beijsterveldt CE, Molenaar PC, de Geus EJ, Boomsma DI (1996). Heritability of human brain functioning as assessed by electroencephalography. American Journal of Human Genetics.

[b35-arh-31-3-238] Von Stein A, Sarnthein J (2000). Different frequencies for different scales of cortical integration: From local gamma to long range alpha/theta synchronization. International Journal of Psychophysiology.

[b36-arh-31-3-238] Wang JC, Hinrichs AL, Stock H (2004). Evidence of common and specific genetic effects: Association of the muscarinic acetylcholine receptor M2 (CHRM2) gene with alcohol dependence and major depressive syndrome. Human Molecular Genetics.

[b37-arh-31-3-238] Yordanova J, Kolev V (1996). Brain theta response predicts P300 latency in children. Neuroreport.

